# Supplementary material for: Establishment and evaluation of a circAdpgk-0001 knockdown method using CRISPR–Cas13d RNA-targeting technology
Source: PeerJ. 2025 Oct 1;13:e20123. doi: 10.7717/peerj.20123 (PMC12495950; doi:10.7717/peerj.20123)
Supplement: Supplemental Information 1 [file peerj-13-20123-s001.docx]

**Supplementary Table Different sequences of siRNAs**

| siRNA | Sequences（5’-3’） |
| --- | --- |
| siRNA-1- sense  siRNA-1- antisense  siRNA-2- sense  siRNA-2- antisense  siRNA-3- sense  siRNA-3- antisense  siRNA-4- sense  siRNA-4- antisense  siRNA-5- sense  siRNA-5- antisense  siRNA-6- sense  siRNA-6- antisense  si-NC- sense  si-NC- antisense | ACAGGCGUUGACUCUUUAATT  UUAAAGAGUCAACGCCUGUTT  GCGUUGACUCUUUAAGUCUTT  AGACUUAAAGAGUCAACGCTT  UUGACUCUUUAAGUCUGUGTT  CACAGACUUAAAGAGUCAATT  CACAGACTTAAAGAGTCAA  TTGACTCTTTAAGTCTGTG  GACTTAAAGAGTCAACGCC  GGCGTTGACTCTTTAAGTC  TTAAAGAGTCAACGCCTGT  ACAGGCGTTGACTCTTTAA  UUCUCCGAACGUGUCACGUTT  ACGUGACACGUUCGGAGAATT |
