# Supplementary material for: Establishment and evaluation of a circAdpgk-0001 knockdown method using CRISPR–Cas13d RNA-targeting technology
Source: PeerJ. 2025 Oct 1;13:e20123. doi: 10.7717/peerj.20123 (PMC12495950; doi:10.7717/peerj.20123)
Supplement: Supplemental Information 2 [file peerj-13-20123-s002.docx]

Supplementary Table 2 Primer pairs used in the current study

| Genes or circRNA | | Primer sequences |
| --- | --- | --- |
| GAPDH  α-SMA  Collagen I  circ-Adpgk_0001 | Forward: 5’- TTCACCACCATGGAGAAGGC -3’  Reverse: 5’- GGCATGGACTGTGGTCATGA -3’  Forward: 5’- TGACGCTGAAGTATCCGATAGA -3’  Reverse: 5’-CGAAGCTCGTTATAGAAAGAGTG -3’  Forward: 5’- CGCCATCAAGGTCTACTGC -3’  Reverse: 5’-ACGGGAATCCATCGGTCA -3’  Forward: 5’-TCACGCCAGAGATTACCACA -3’  Reverse: 5’- GACCTTTCATGACATTGCCCA -3’ | |
