# Supplementary figures and images for: Establishment and evaluation of a circAdpgk-0001 knockdown method using CRISPR–Cas13d RNA-targeting technology
Source: PeerJ. 2025 Oct 1;13:e20123. doi: 10.7717/peerj.20123 (PMC12495950; doi:10.7717/peerj.20123)

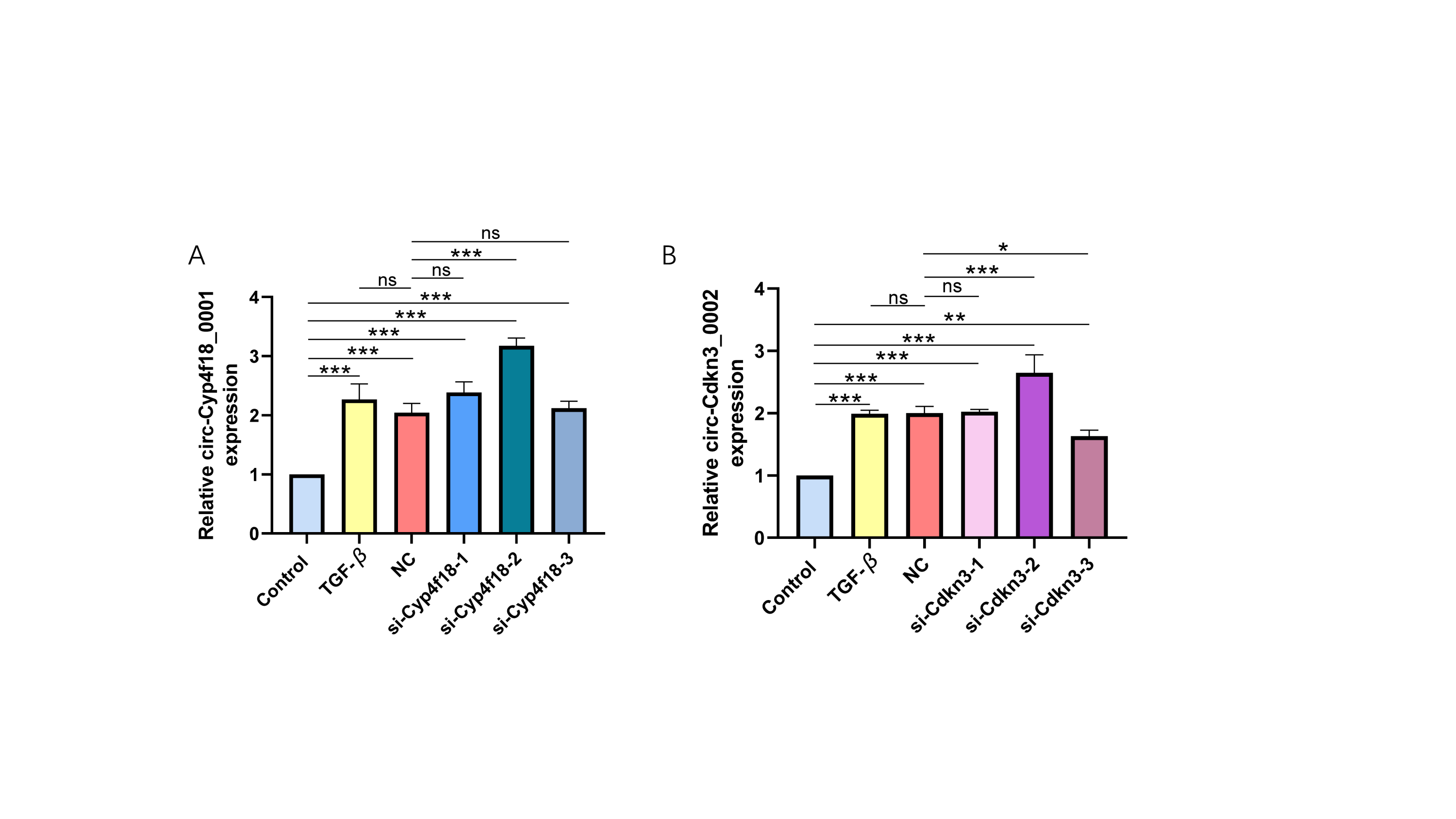

Supplement: Supplemental Information 3 — (A) The knockdown efficiency of circ-Cyp4f18_0001 by different siRNAs (n = 3). (B) The knockdown efficiency of circ-Cdkn3_0002 by different siRNAs (n = 3). *p < 0.05, **p < 0.01, ***p < 0.001. [file peerj-13-20123-s003.png]
